# Somatostatin receptor expression, tumour response, and quality of life in patients with advanced hepatocellular carcinoma treated with long-acting octreotide

**DOI:** 10.1038/sj.bjc.6603325

**Published:** 2006-09-05

**Authors:** J Cebon, Australasian Gastro-Intestinal Trials Group (AGITG) AG0001H Investigators

**Affiliations:** 1 AGITG Trial Coordinating Centre, NHMRC Clinical Trials Centre, University of Sydney, Locked Bag 77, Camperdown, NSW 1450, Australia

**Keywords:** hepatocellular carcinoma, octreotide, scintigraphy, receptors, somatostatin, toxicity, feasibility study

## Abstract

Octreotide may extend survival in hepatocellular carcinoma (HCC). Forty-one per cent of HCCs have high-affinity somatostatin receptors. We aimed to determine the feasibility, safety, and activity of long-acting octreotide in advanced HCC; to identify the best method for assessing somatostatin receptor expression; to relate receptor expression to clinical outcomes; and to evaluate toxicity. Sixty-three patients with advanced HCC received intramuscular long-acting octreotide 20 mg monthly until progression or toxicity. Median age was 67 years (range 28–81 years), male 81%, Child–Pugh A 83%, and B 17%. The aetiologies of chronic liver disease were alcohol (22%), viral hepatitis (44%), and haemochromatosis (6%). Prior treatments for HCC included surgery (8%), chemotherapy (2%), local ablation (11%), and chemoembolisation (6%). One patient had an objective partial tumour response (2%, 95% CI 0–9%). Serum alpha-fetoprotein levels decreased more than 50% in four (6%). Median survival was 8 months. Thirty four of 61 patients (56%) had receptor expression detected by scintigraphy; no clear relationship with clinical outcomes was identified. There were few grade 3 or 4 toxicities: hyperglycaemia (8%), hypoglycaemia (2%), diarrhoea (5%), and anorexia (2%). Patients reported improvements in some symptoms, but no major changes in quality of life were detected. Long-acting octreotide is safe in advanced HCC. We found little evidence of anticancer activity. A definitive randomised trial would identify whether patients benefit from this treatment in other ways.

Hepatocellular carcinoma (HCC) is a major cause of cancer death and morbidity worldwide (Johnson, 1996) It is frequently complicated by pre-existing chronic liver disease, limiting therapeutic options. While early disease can be managed by resection or local ablation, decreased hepatic reserve may limit the options for surgery or systemic therapy. Furthermore, response rates for systemic therapy are low: no single agent has a response rate higher than approximately 20%, and none improves survival (Leung and Johnson, 2001). Multiagent therapy using *cis*-platinum, doxorubicin, 5-fluorouracil, or interferon-alpha may show higher response rates (Leung *et al*, 1999). However, because patients with HCC frequently have advanced liver disease and associated comorbidities, they are often unable to tolerate intensive and toxic treatment. On the basis that some HCCs have hormone receptors, hormonal approaches (including tamoxifen in a variety of doses) have been evaluated; however, none of these has shown a survival advantage over best supportive care (Chow *et al*, 2002).

A small randomised trial conducted by Kouroumalis *et al* (1998) suggested that survival was longer in a group treated with short-acting octreotide than in an untreated control group (median survival 13 vs 4 months, 12-month survival 56 vs 13%, *P*=0.002). High-affinity somatostatin receptors capable of binding octreotide are present in approximately 40% of HCC, and not in adjacent liver. However, the numbers of high-affinity receptors are lower than reported for neuroendocrine tumours (Reubi *et al*, 1999). Somatostatin is a potent inhibitor of several growth factors and angioneogenesis (Florio *et al*, 2003); it also has immune-modulating properties (Lambrecht, 2001). Neuroendocrine and epithelial tumour cell growth can be inhibited *in vitro* and *in vivo* by the semisynthetic somatostatin analogue, octreotide (Lamberts *et al*, 1990). The long-acting depot form of octreotide (sandostatin long-acting release (LAR)) is formulated in microspheres, enabling treatment to be delivered by monthly intramuscular (i.m.) injection. It is currently used for the treatment of acromegaly (Freda, 2002) and functional neuroendocrine tumours (de Herder and Lamberts, 2002). Little information is available about its elimination kinetics in cirrhosis, although the short-acting form has been studied (Jenkins *et al*, 1998).

In patients with portal hypertension, octreotide has been used extensively in the management of acute variceal bleeding and other complications, where it reduces portal blood pressure in patients with poor liver function (Jenkins *et al*, 1997). Although the benefit observed by Kouroumalis *et al* (1998) may be a direct anticancer effect mediated by specific receptors on HCC cells, its effects on portal hypertension may also reduce the frequency of variceal bleeding. Octreotide therefore has the potential to affect survival by more than one mechanism.

We undertook a clinical trial to evaluate octreotide LAR in patients with unresectable HCC. The aims of this study were to assess the feasibility, safety, and activity of octreotide LAR in patients with advanced HCC before considering a definitive randomised phase III trial. Because clinical outcomes could potentially depend on somatostatin receptor status, we sought to evaluate the relationship between somatostatin receptor expression, objective response rate, and survival. The utility of noninvasive scanning as a predictor of response was examined by correlating octreotide scintigraphic scanning and biopsy data with survival. Receptor expression was evaluated by immunohistochemistry (IHC) and external scanning using labelled ligand (octreoscan). Since the study population was largely composed of people with impaired liver function and portal hypertension, plasma pharmacokinetic studies were also performed to assist in the overall assessment of the feasibility of using octreotide LAR in these patients.

## PATIENTS AND METHODS

All patients had unresectable HCC, diagnosed either by histology or by the combination of typical findings on imaging and alpha-fetoprotein level (AFP) >500 IU ml^−1^. World Health Organisation performance status was 0–2 and no previous treatment with octreotide for HCC was permitted. Exclusion criteria included white blood count <2.0 × 10^9^ l^−1^, platelets <50 × 10^9^ l^−1^, haemoglobin <10.0 g l^−1^, serum creatinine >0.15 mmol l^−1^, bilirubin >50 mol l^−1^, albumin <25 g l^−1^, and AST and ALT >5 times the upper limit of normal. Patients were also excluded if the prothrombin ratio (INR) was >2.0, if they were receiving concurrent antitumour treatment for HCC, or if they had uncontrolled ascites requiring paracentesis within 4 weeks, variceal bleeding in the previous month, prior radiation therapy to the only evaluable site of disease, or Child–Pugh class C cirrhosis. All patients gave informed consent and the protocol was approved by the institutional review board of each participating institution.

Treatment consisted of octreotide LAR 20 mg by deep i.m. injection every 28 days. The planned duration of treatment was 12 months in the absence of disease progression, unacceptable toxicity, or withdrawal at the patient's or doctor's discretion. An extension phase allowed treatment to continue beyond 12 months on an individual basis at the discretion of the principal investigator.

Before starting treatment, all subjects had a complete medical history and physical examination, chest X-ray, and imaging to fully define the extent of disease. Triple-phase spiral computed tomography (CT) was recommended for imaging the liver. Blood tests included full blood count, tests of liver function (serum albumin, alkaline phosphatase, total bilirubin, AST, and ALT), coagulation (INR and APTT), blood sugar, AFP, and tests to evaluate the aetiology of liver disease (HBsAg and anti-HCV). Blood was also sampled for octreotide pharmacokinetics and assessment of chromogranin A levels before the 4th and 7th doses. All patients had an octreotide scintigraphy scan before commencing treatment.

Tumour biopsy for receptor analysis was optional, depending on the availability of suitable tissue. Where available, archival material was assessed for receptor expression by IHC at a central laboratory.

Subjects were seen monthly for clinical review, blood tests, and administration of the study drug. Response was assessed using the RECIST (response evaluation criteria in solid tumours) criteria (Therasse *et al*, 2000). Radiological assessment was undertaken 3-monthly and any partial or complete responses were confirmed 1 month later.

Health-related quality of life (HRQL) was self-rated by patients each month before their clinical review using two instruments: the FACT-Hep (Heffernan *et al*, 2002) and the ‘Patient Disease And Treatment Assessment’ form (Pt DATA Form). After the first month of treatment, patients also rated how aspects of their HRQL had changed, using a series of transition scales referred to here as the subjective Patient Benefit Form. We decided *a priori* to focus on changes in HRQL at 1 month because the number of evaluable patients was expected to fall quickly from then on.

The FACT-Hep (Heffernan *et al*, 2002) is a validated 45-item self-rated questionnaire, incorporating the FACT-G questionnaire (27 items) (Cella *et al*, 1993), which covers multiple general aspects of HRQL, and an 18-item module specific for hepatobiliary cancer. Scores are calculated for four domains from the FACT-G: physical, social–family, emotional, and functional well-being. In addition, a total score including the hepatobiliary-specific items is calculated (Cella, 1997).

The Patient DATA Form is a simple, pragmatic, patient-rated instrument designed to measure aspects of HRQL that are relevant to people with advanced cancer. Its development is reported elsewhere (Nowak *et al*, manuscript in preparation). It assesses 24 aspects of HRQL using simply worded items listed on a single page: 16 physical and emotional symptoms of cancer rated on a numeric scale from 0 (no trouble at all) to 10 (worst I can imagine) and eight aspects of well-being rated from 0 (worst possible) to 10 (best possible) (Appendix A).

The Patient DATA Form is designed to be rapidly and easily interpreted: there is no scoring or aggregation procedure. Items are arranged in two blocks: symptoms and dysfunctions (0 on left, 10 on right) where high scores reflect worse quality of life or more severe symptoms; and aspects of well-being (10 on left, 0 on right) where high scores reflect better well-being and quality of life. Troublesome aspects stand out by being circled on the right side of the page.

The Patient Benefit Form is a health-transition scale that rates changes in the same aspects assessed with the Patient DATA Form using a similar simple, one-page format. Patients rated how each symptom or function has changed since their first injection 4 weeks previously using a five-point Likert scale: much better, a little better, the same, a little worse, or much worse.

### Pharmacokinetics

Samples were taken immediately before the 4th and 7th doses of octreotide (weeks 12 and 24 of treatment). These time points were selected in order to assess trough octreotide plasma concentrations at steady state after three doses and to determine potential octreotide accumulation in this cirrhotic population with more continued dosing. Additional samples were also collected in some cases. Plasma octreotide levels were determined by radioimmunoassay kindly performed by M Rouilly (Novartis Pharma AG, Basel, Switzerland). The lower limit of quantification was 50 pg ml^−1^ for a 50 *μ*l plasma sample.

### Octreotide scintigraphy

^111^In-pentetreotide, 130–220 MBq, was administered intravenously. Images were acquired at all study sites with a standardised imaging protocol. All images were acquired on dual-headed gamma cameras using medium-energy parallel-hole collimators. Anterior and posterior planar images (15 min image^−1^) were acquired over the abdomen and at other sites of known disease 24 h after injection. Additional whole-body or SPECT images were acquired after 4 or 24 h in some patients.

Two experienced nuclear medicine physicians assessed the images, blinded to each other's interpretation. Tracer uptake in the primary tumour and metastases was determined relative to normal liver. Abdominal CT scan reports were used for tumour localisation. Uptake at the tumour site was scored as negative, clear but faint, moderate, or intense. Disagreement between reviewers was resolved by consensus.

### Immunopathology

Archival paraffin-embedded tissue was stained for somatostatin receptor, type SSTR2A, using rabbit polyclonal antiserum SS800 (Jomar Diagnostics, Adelaide, Australia) raised against amino-acid sequences 355–369 (ETQRTLLNGDLQTSI) of human, rat, and mouse SSTR2A receptor coupled to KLH. This antibody recognises the C-terminus of human, rat, and mouse SSTR2A receptor. Specificity of antibody binding was determined by blocking with cognate peptide. Positive control staining was determined using normal pancreas, islet cell, and other neuroendocrine tumours, where cytoplasmic and membrane staining of normal pancreatic islets was observed. Three-micron sections were pretreated with 10 mM sodium citrate, pH 6.0, for 2 min. Endogenous peroxidase was blocked with 3% hydrogen peroxide for 10 min. Antiserum was applied at 1 : 2000 in 50 mM Tris-HCl, pH 7.6, 0.05% for 30 min at room temperature. Chromogen was DAB (DakoCytomation, Sydney, Australia).

### Chromogranin A

Plasma chromogranin A levels were measured to see whether this marker of neuroendocrine differentiation provided a useful correlation with receptor expression, thereby serving as a simple blood test for receptor-bearing tumours. The levels were assayed by Network Pathology (Melbourne, Australia), using the Chromogranin A ELISA kit (DakoCytomation, Sydney, Australia).

### Statistical methods

The sample size of 63 was designed to give >80% power to detect an 8-month difference in median survival comparing the 40% patients expected to have a positive octreoscan *vs* the remainder expected to have a negative octreoscan (estimated survivals 12 and 4 months, respectively, as per the treatment and control arms in the Kouroumalis study). Survival times were calculated from the day of registration. Survival curves were calculated using the method of Kaplan–Meier. The log-rank test was use to compare the relationship between receptor expression by scintigraphy and survival duration. Proportions were compared using *χ*^2^ tests. Quality-of-life results were described and analysed with simple descriptive and comparative methods. For each item in the Patient Benefit Form, we recorded the proportions of patients reporting that each aspect was much better, a little better, the same, a little worse, or much worse than before starting treatment, and used *χ*^2^ tests to compare the proportions of patients who reported feeling better *vs* those who reported feeling worse. For the Patient DATA Form and FACT-Hep, we used Wilcoxon's rank-sum tests for paired data to compare baseline and 1-month scores for each domain. Responses from the Patient Benefit Form were compared with change scores from the Patient DATA Form using Spearman's rank-correlation coefficient.

## RESULTS

The study accrued 63 patients with otherwise untreatable HCC between April 2001 and January 2002. Study sites are listed in Appendix A. The patients' baseline characteristics are shown in Table 1. The median age was 67 years (range 28–81 years) and most of them were male. Most had good performance status. Eleven had Child–Pugh class B cirrhosis and 52 had well-compensated disease (Child–Pugh class A). Viral hepatitis was the main cause of cirrhosis, with 28 patients (44%) having evidence of hepatitis B or C infection. A small number of patients had cirrhosis from other causes. Approximately half had received other treatment for HCC.

### Administration of treatment

Four patients (6%) did not receive any octreotide because their disease progressed so rapidly they were unable to start treatment. These patients were not eligible for analysis of response or toxicity, but are included in the survival analysis. Three patients (5%) received one dose, 12 (19%) two doses, 10 (16%) three doses, 10 (16%) four doses, seven (11%) five doses, four (6%) six doses, five (8%) seven doses, and seven (18%) eight doses or more. The main reason for stopping treatment was disease progression in 19 patients (30%). A further five patients (8%) withdrew and nine (14%) died during treatment, all from progressive disease. Treatment was stopped because of adverse effects (cramps) in only one case.

### Tumour response

Objective tumour response data for all 63 patients are summarised in Table 2. Reductions in AFP were accompanied by imaging evidence of partial response in one patient (2%, 95% CI 0–9%), stable disease in one patient, and progressive disease in two patients.

### Survival

**Figure 1:**
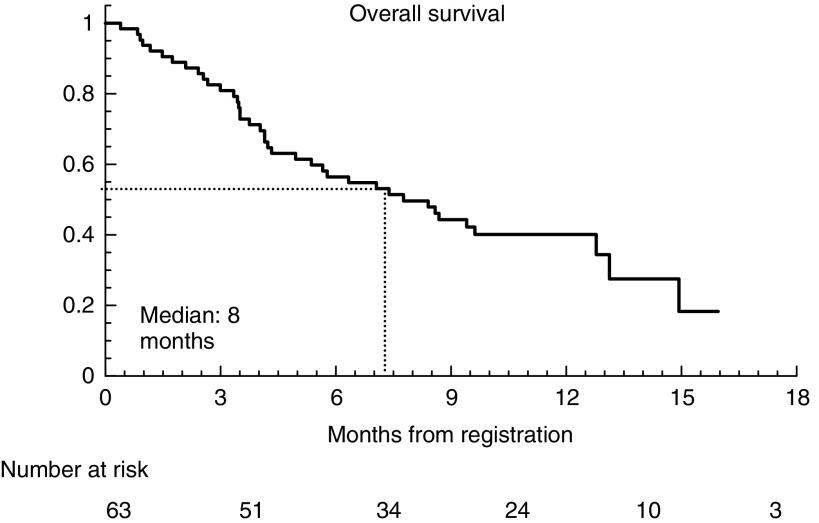
Overall survival.

**Figure 2:**
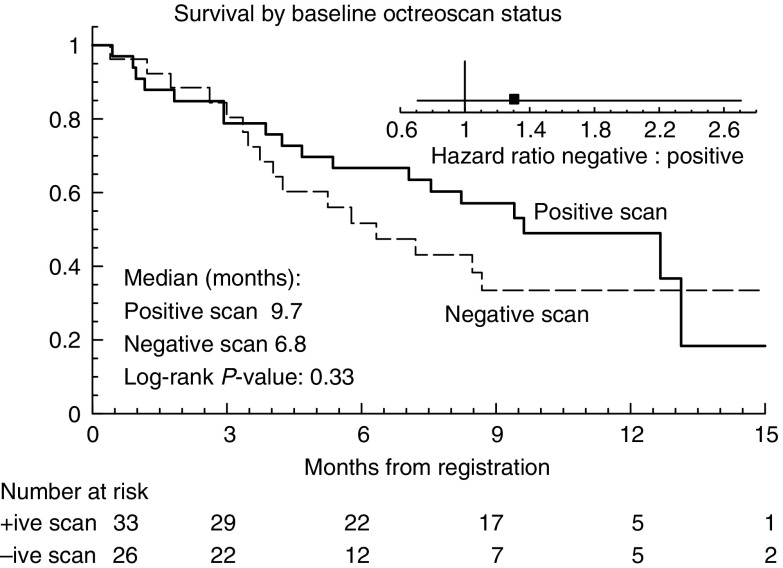
Survival, by baseline octreotide scintigraphy status. The small graph shows the hazard ratio of the negative group (−) compared with the positive group (+).

Survival status is shown in Table 3 and Figure 1. After a median follow-up of 12 months, the median survival was 8 months (range 1–25 months), and five patients remain on treatment 21–25 months after starting. There was no statistically significant relationship between receptor expression by octreotide scintigraphy and survival duration (*P*=0.33) (Figure 2).

Treatment was well tolerated. Table 4 shows the main toxicities, including diarrhoea, abdominal cramping, and alterations in blood sugar (chiefly hyperglycaemia). Most toxicities were grade 1 or 2; the only grade 3 or 4 toxicities were diarrhoea, alterations in blood sugar, and anorexia. Diarrhoea is a common symptom in patients with advanced HCC even in the absence of any treatment.

### Adverse events

Most of the adverse events were attributable to chronic liver disease and its complications (Table 5). All deaths were attributable to progressive HCC. Two adverse events had unrelated causes (incarcerated inguinal hernia and an abscess).

### Gastrointestinal bleeding

With a total follow-up approaching 4000 patient-months, gastrointestinal bleeding occurred in four of 63 patients. In one patient, this was from peptic ulcer disease and in the others was from varices.

### Pharmacokinetics, receptors, and markers

Pharmacokinetic evaluation was performed on 62, 41, 25, and 7 patients at baseline and after 3, 6, and 9 months of treatment, respectively. The mean trough plasma concentration at 3 months was 2205 pg ml^−1^ (range 678–6166 pg ml^−1^). After 6 months, the mean concentration of plasma octreotide was 3377 pg ml^−1^ (range 956–14 290 pg ml^−1^), and after 9 months, 3238 pg ml^−1^ (1307–9926 pg ml^−1^). Evaluation of those patients who remained on the study for 6 months or more showed no evidence of accumulation of octreotide in plasma. Although plasma concentrations fluctuated, they were generally within the range 1000–3000 pg ml^−1^.

Serum AFP was elevated in 50 patients (79%). In four of these it fell, individually, from 578 to 2, from 1600 to 2, from 48 to 23, and from 1368 to 56 IU ml^−1^. Serum for chromogranin A estimation and pharmacokinetics was available from 59 patients (94%), and tissue was available for IHC analysis of somatostatin receptors from 20 samples from 19 patients (44%); one patient had two tumours.

### Octreotide scintigraphy

**Figure 3:**
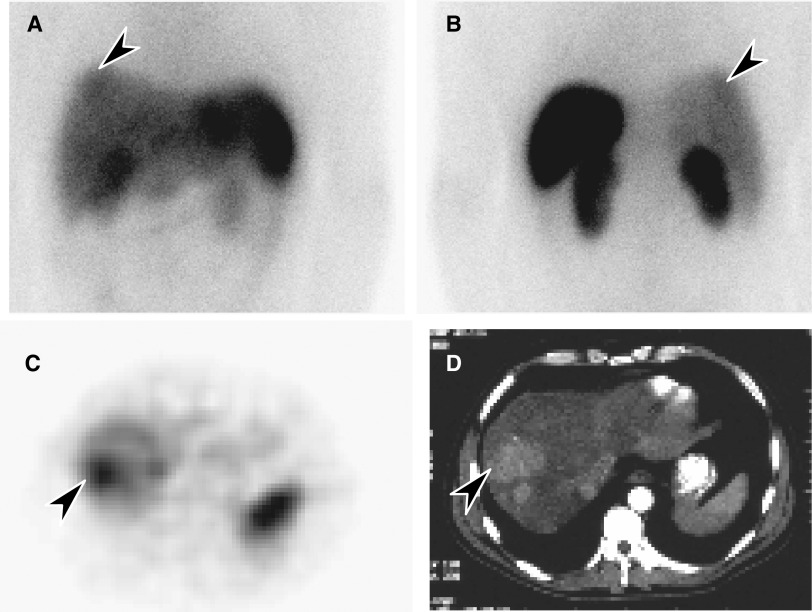
(**A**) Octreotide scintigraphy (4-h image) showing uptake in an HCC in the dome of the right lobe of the liver (arrow): anterior image; (**B**) posterior image; normal octreotide uptake in spleen and kidneys; (**C**) SPECT transaxial section through the upper abdomen showing increased uptake in the HCC (arrow); and (**D**) the hepatoma in a corresponding CT scan slice (arrow).

**Figure 4:**
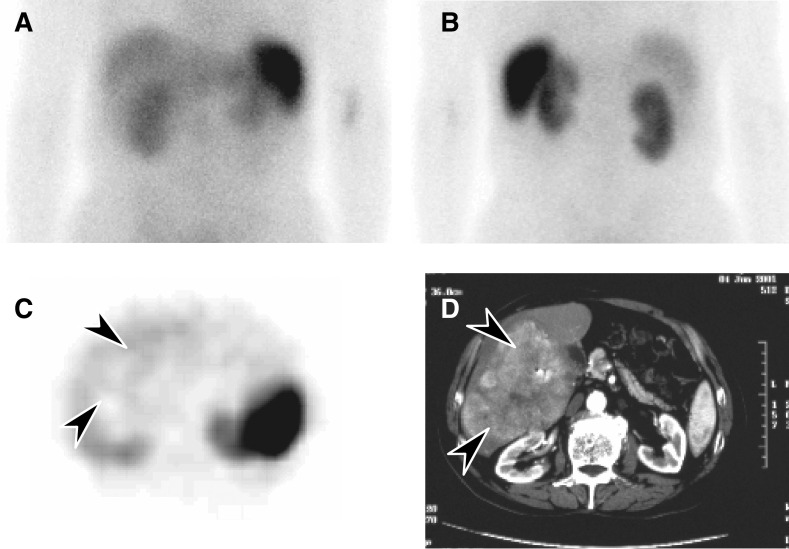
(**A**) Octreotide scintigraphy (4-h image) showing no evidence of uptake in an HCC in the right lobe of the liver: anterior image; (**B**) posterior image; (**C**) SPECT transaxial section through the upper abdomen showing reduced uptake in the HCC (arrows) compared with normal liver; and (**D**) the HCC in a corresponding CT scan slice (arrows).

Sixty-one patients had octreotide scans. Table 6 shows the relationship between the degree of uptake in lesions as assessed by scintigraphy and the detection of tumour by conventional imaging. Overall, 34 of 61 patients (56%) had positive scintigrams. Uptake was clear but faint in the majority of patients. Examples of octreotide uptake are shown in Figures 3 and 4. In general, the test was less sensitive than CT scanning in identifying HCC; however, it provided a noninvasive indication of the presence or absence of somatostatin receptors. In some instances (Figure 4), the tumour area showed less uptake than normal liver.

### Immunohistochemistry for somatostatin receptors

Positive controls (pancreatic islets, neuroendocrine tumours) showed clear staining. In the 20 tissue samples that were suitable for IHC analysis, there were not enough receptors for meaningful staining with the antiserum SS800, despite considerable efforts to optimise antibody staining.

### Chromogranin A

**Figure 5:**
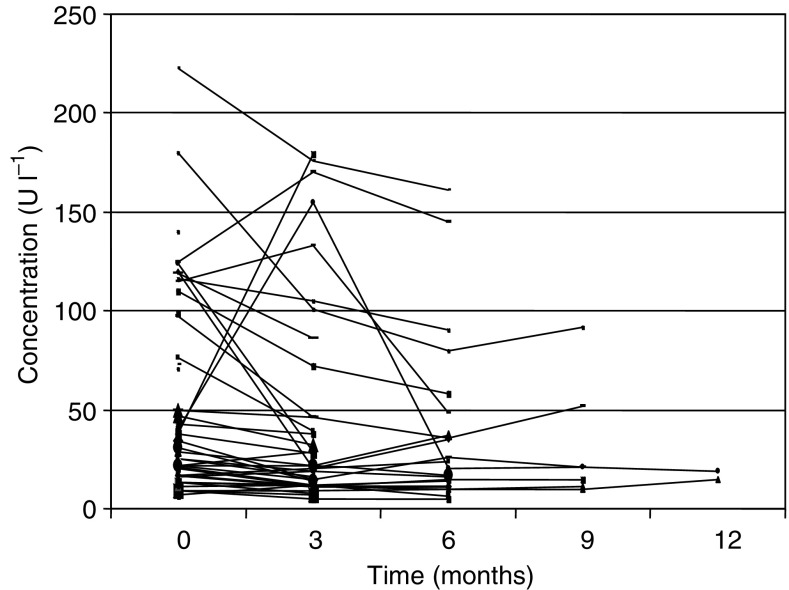
Chromogranin A levels over time in each patient.

Figure 5 shows chromogranin A levels. We hypothesised that patients with high levels might be those with tumours, which displayed features of neuroendocrine differentiation and therefore hormone receptors. In turn, these might be those most likely to respond to therapy with the somatostatin ligand. We therefore analysed the relationships among chromogranin levels, scintigraphy results, and clinical outcomes. There was no clear relationship between scan positivity and chromogranin A levels. Similarly, there was no relationship between serum levels of chromogranin A and survival.

### Quality of life

**Figure 6:**
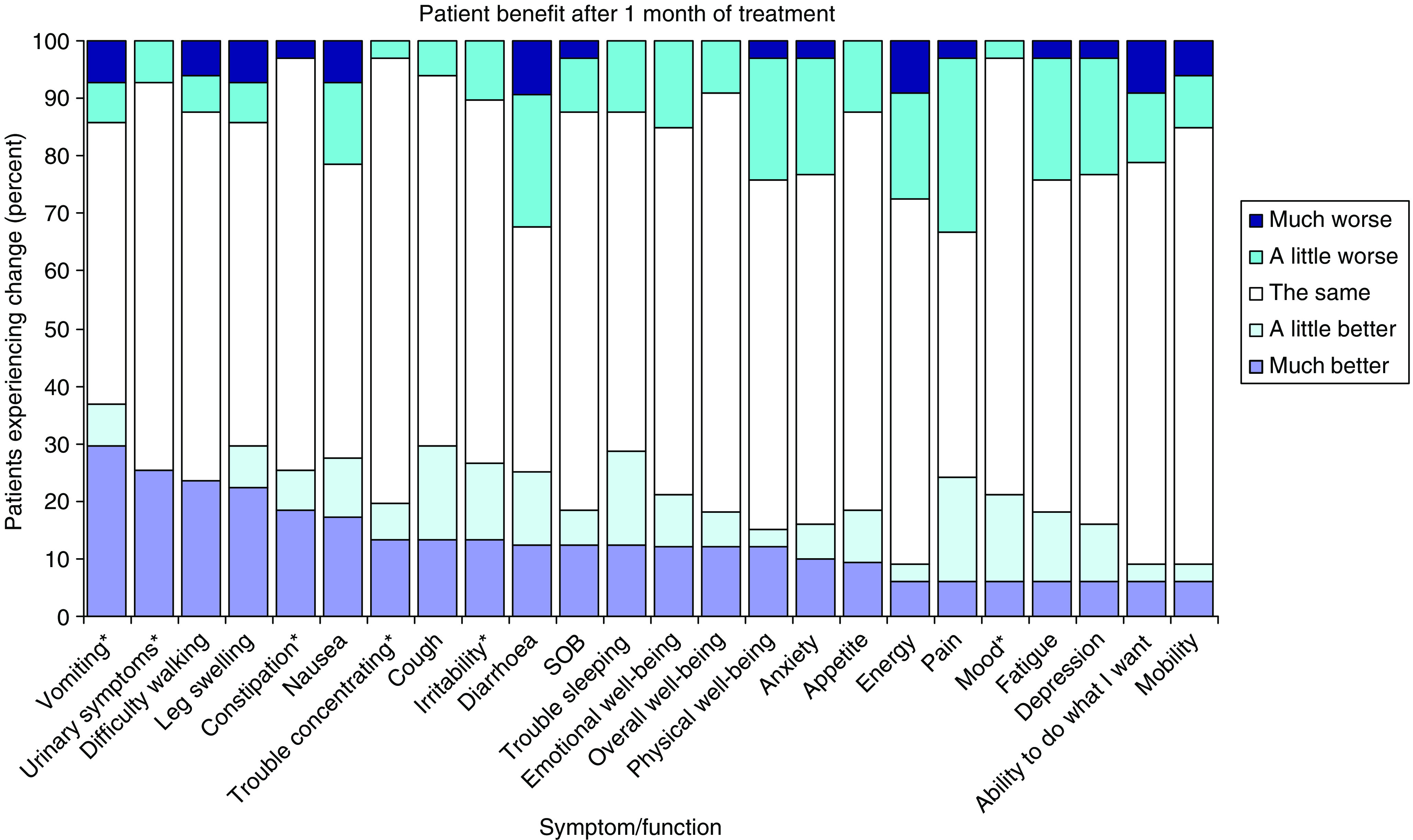
Subjective HRQL status reported on the PBF after 1 month of treatment. ^*^P<0.05.

Most patients had adequate English skills to complete the HRQL forms (47 patients, 75%) and most did so at baseline (46 patients), but completing the forms decreased over follow-up, as expected. Fatigue, anxiety, pain, and insomnia were the symptoms rated as most severe at baseline. After 1 month of treatment, significantly more patients reported improvements than deteriorations on the Patient Benefit Form in vomiting, urinary symptoms, constipation, cough, irritability, and mood (Figure 6). Differences between ratings of these aspects at baseline and after 1 month, on the Pt DATA form, were in the same direction as the ratings from the Patient Benefit Form, but were not statistically significant, and correlations between the two instruments were modest (Spearman's *r*<0.3). There were no significant changes in the FACT-Hep between baseline and 1 month. More detailed analysis of the HRQL data will be reported elsewhere.

## DISCUSSION

The main finding of this study was that octreotide LAR was well tolerated in patients with advanced HCC – a population with cirrhosis and chronic liver disease. Although there were few objective tumour responses (one patient with AFP response plus partial response on imaging, one patient with AFP response plus stable disease on imaging), 16 patients (25%) had stable disease or better for at least 3 months. Forty per cent had only 1–3 injections of octreotide LAR. It takes about 3 months for steady-state levels to be built up, so these patients were technically underdosed. The median survival, 8 months, was within the range of published series (CLIP Group, 1998; Kouroumalis *et al*, 1998; Chow *et al*, 2002; Dimitroulopoulos *et al*, 2002; Samonakis *et al*, 2002; Yuen *et al*, 2002). Receptor expression was identified by octreotide scintigraphy in 34 of 61 patients (56%). There was no clear relationship between scan positivity and survival. Approximately 25% of patients reported improvements in some aspects of HRQL while on octreotide.

Unresectable HCC commonly occurs in patients with advanced chronic liver disease and is difficult to treat. Comorbidities and poor performance status limit the use of cytotoxic chemotherapy, so identifying less toxic approaches is important. The small randomised trial reported by Kouroumalis in 1999 showed an implausibly large effect of octreotide on survival (Kouroumalis *et al*, 1998). It is impossible to tell from this trial whether this effect on survival was real, and if so, whether it was due to anticancer effects or other effects such as reduction in portal pressure. Subsequent studies with long-acting octreotide analogues have shown less benefit (Raderer *et al*, 2000; Samonakis *et al*, 2002) or no benefit (Yuen *et al*, 2002). In a recent study, patients selected for treatment on the basis of scan positivity were treated with octreotide and, when compared with a control population in a nonrandomised manner, had better outcomes in terms of survival and quality of life (Dimitroulopoulos *et al*, 2002). Nonetheless, this question will remain open until a definitive confirmatory randomised trial is performed.

Safety is important for people with comorbidities. There is limited information available about the elimination kinetics of octreotide in cirrhosis. Jenkins *et al* (1998) have demonstrated prolonged half-life and reduced clearance in patients with cirrhosis and suggested that dosage regimens should be modified in such patients to avoid accumulation of the analogue in the blood, which may result in undesirable side effects or toxicity. Since pharmacokinetics may be altered in patients with cirrhosis, it was considered necessary to determine whether liver impairment predisposes to toxicity and whether or not this dose was tolerated in this patient population. A somewhat reduced dose of 20 mg month^−1^ was arbitrarily selected to evaluate toxicity in this largely cirrhotic population. At this dose, plasma concentrations were achieved in the range 1000–3000 pg ml^−1^. These levels are similar to those typically seen in noncirrhotic patients. For example, a 30 mg i.m. dose resulted in plasma concentrations of 1682±174 pg ml^−1^ in a series of six patients with acromegaly (Stewart *et al*, 1995). Although sufficient to suppress secretory function in acromegaly or carcinoid syndrome, it is unclear whether these concentrations are sufficient for therapeutic impact in HCC since a dose–response relationship has not been established for octreotide as an anticancer agent.

Octreotide treatment was well tolerated. The most common side effects were mild gastrointestinal symptoms and asymptomatic abnormalities of blood sugar levels. No serious adverse events were attributed to octreotide.

Most patients with advanced HCC have a dismal prognosis (Leung and Johnson, 2001). However, a treatment that benefits only a subset of patients may still be clinically worthwhile. The very different outcomes in the randomised trials by Kouroumalis and Yuen probably reflect differences in patient selection (Kouroumalis *et al*, 1998; Yuen *et al*, 2002). The Yuen study had subjects with more advanced disease, a higher proportion with viral hepatitis, and a median survival of only 2 months. Our study population was more similar to the Kouroumalis study population (Table 4) and our median survival of 8 months lies midway between those of the two arms of the Kouroumalis study: 13 months in the octreotide arm and 4 months in the placebo arm.

As HCC can express high-affinity receptors for somatostatin (Reubi *et al*, 1999), it was important to determine whether a relationship existed between the presence of somatostatin receptors and clinical outcomes. We found that octreotide uptake was highly variable, ranging from less than the uptake of normal liver to high or intense (Table 6), and this variability was even seen within an individual (Figure 3). Few patients had intense uptake (10%), consistent with the observation that receptor numbers in HCC tend to be lower than those in neuroendocrine tumours (Reubi *et al*, 1999). While octreotide scintigraphy was less helpful than CT scanning for identifying anatomical sites of tumour, its value may be in providing functional information about these tumours, particularly evidence of neuroendocrine differentiation. Confirmation of this using another technique is warranted. As fresh tissue was not consistently available, it was not possible to perform radioligand labelling studies to confirm this *in vitro* or at a microscopic level. Receptor analysis by IHC was performed on fixed tissues, but we were not able to reliably detect receptors in tissue sections. We were therefore unable to show a relationship between the antibody method and scintigraphy results.

Although HCCs do not typically show neuroendocrine differentiation on histological examination, measurement of serum chromogranin A levels was undertaken on the basis of a recent report that levels can be elevated in approximately one-third of cases (Leone *et al*, 2002). It was proposed as both a potential prognostic marker and a marker of neuroendocrine differentiation. In our series, six of 59 patients had levels 10 U l^−1^ or less (reference range 2–8 U l^−1^), 35 had levels of 10–50 U l^−1^, and 20 had levels higher than 50 U l^−1^. Although most patients progressed on radiological criteria, chromogranin A levels decreased in 15 of 59 patients (25%) and remained stable in 25 (40%), increasing in a sustained manner in only two patients (3%). We found no evidence that receptor expression or neuroendocrine features were associated with either scan positivity or clinical outcomes: chromogranin A therefore did not appear to be a useful marker for identifying a subset of tumours more likely to respond, or as a marker for monitoring clinical progress, with the proviso that the small sample size did not provide enough statistical power to detect other than large effects.

Octreotide lowers portal pressure, and is routinely used to treat patients with variceal bleeding. Since prophylactic octreotide can prevent bleeding in high-risk patients (Jenkins *et al*, 1997), octreotide LAR might reduce morbidity and mortality through this mechanism. In our trial, gastrointestinal bleeding occurred in four patients (one peptic ulcer, three varices) with a total follow-up approaching 4000 patient-months. It is impossible to determine the effect of octreotide LAR on the rate of complications from portal hypertension without a randomly allocated control group; however, the rate in our study seems low compared with other series (Akanuma *et al*, 2002).

Although more patients rated improvements than deteriorations for some symptoms, these ratings were not strongly correlated with receptor status scores, and it is unclear whether these represent an effect of treatment. However, the fact that many patients reported improvements may partly explain the clinical impression of benefit in some patients. Determining the significance and cause of these changes requires a randomly allocated control group. Our findings support the feasibility and importance of incorporating measures of HRQL in future trials in advanced HCC.

This study has shown that octreotide LAR is well tolerated and may benefit some patients with HCC. We were unable to define, from octreotide scintigraphy or chromogranin A analyses, a subgroup more likely to benefit. There was little objective evidence of antitumour activity. A phase III study is advocated and indeed warranted given the conflicting findings with previous studies. However, careful attention should be paid to the selection of the primary end point and dose selection in the design of such a study. The potential impact on quality of life, survival, and cirrhosis-related morbidity, particularly variceal bleeding, justifies the performance of such a trial.

## Figures and Tables

**Table 1:** Patient characteristics

**Characteristic**	***n***	**%**
*Sex*		
Male	51	81
Female	12	19
		
*WHO performance status*		
0	20	32
1	30	48
2	13	21
		
*Cirrhosis: Child–Pugh class*		
A	52	83
B	11	17
		
Raised AFP	50	79
		
*Race*		
Caucasian	40	63
Asian	14	22
African	1	2
Polynesian or Melanesian	4	6
Other	4	6
		
*Possible causes of HCC* a		
Alcoholic liver disease	14	22
Hepatitis	40	63
HBsAg and no antibody to HCV	16	25
Antibody to HCV and no HBsAg	9	14
HBsAg and antibody to HCV	3	5
No HBsAg or antibody to HCV	6	10
Unclassified	6	10
Haemochromatosis	4	6
Other	7	11
		
*Most recent treatment for HCC*		
No previous therapy	31	49
Surgery	5	8
Chemotherapy	1	2
Local ablation	7	11
Chemoembolisation	4	6
Other	15	24

HBsAg=hepatitis B surface antigen; HCC=hepatocellular carcinoma; HCV=hepatitis C virus; WHO=World Health Organisation.

a Some patients had several causes.

**Table 2:** Tumour responses

**Best response**	***n***	**%**
*Tumour response (RECIST)*		
Complete response	0	0
Partial response^a^	1	2
Stable disease	15	24
Progressive disease	23	37
Unknown	9	13
Not available	15	24
		
Total assessable	63	100
		
*AFP response* a ^,^ b		
>50% reduction^c^	4	7
>50% reduction and no progressive disease^c^	2	3
		
Total assessed	59	100

AFP=alpha-fetoprotein; RECIST=response evaluation criteria in solid tumours.

a Confirmed on two consecutive assessments at least 4 weeks apart.

b Baseline AFP level needed to be more twice the upper limit of normal to qualify as a response.

c These two AFP responses without progressive disease are also counted in the row above.

**Table 3:** Survival status at March 2003

**Patient status**	***n***	**%**
Dead (tumour progression)	48	76
Dead (other causes)^a^	2	3
Alive, still on study treatment	5	8
Alive, not on study treatment	8	12
		
Total	63	100

a One, pneumonia; one, sepsis.

**Table 4:** Toxicity: numbers of patients with each grade as their most severe during their course of treatment

	**Toxicity grade (NCI)^a^**					
**Type of toxicity**	**0^b^**	**1**	**2**	**3**	**4**	**% with grade 3 or 4**
Diarrhoea	28	22	10	3	0	5
Abdominal cramping	47	9	7	0	0	0
Hyperglycaemia	45	4	9	5	0	8
Hypoglycaemia	60	2	0	0	1	2
Bruising	60	3	0	0	0	0
Alopecia	62	1	0	0	0	0
Pancreatitis	63	0	0	0	0	0
Anorexia	40	14	8	1	0	2
Other	63	0	0	0	0	0

a NCI Common Toxicity Criteria Version 2.

b Grade 0=no toxicity.

**Table 5:** Serious adverse events

**Type of event**	***n***
Gastrointestinal bleeding	4
Ascites	4
Hepatic encephalopathy	1
Liver failure	1
Dyspnoea	1
Brain metastases	1
Diabetes mellitus	1
Axillary abscess	1
Incarcerated hernia	1
	
Total	15

**Table 6:** Octreotide scintigraphy (61 patients) and conventional imaging (CT scanning, 63 patients) compared

			**Number of patients with the given degree of radioactive uptake in the tumour at this site**		
**Site**	**Patients with disease at this site confirmed on CT**	**Patients with positive scintigraphy at this site**	**Clear but faint**	**Moderate**	**Intense**
Bone	3	2	1	0	1
Liver	61	32	18	9	5
Lung	3	0	0	0	0
Other	11	4	3	1	0

CT=computed tomography

**Table A1:** 

**Writing committee**	
Jonathan Cebon	Austin Hospital, Melbourne
Michael Findlay	University of Auckland, Auckland
Carol Hargreaves	NHMRC Clinical Trials Centre, University of Sydney
Martin Stockler	NHMRC Clinical Trials Centre, University of Sydney
Paul Thompson	Auckland Hospital, Auckland
Michael Boyer	Royal Prince Alfred Hospital, Sydney
Stuart Roberts	Alfred Hospital, Melbourne
Aurora Poon	Austin Hospital, Melbourne
Andrew M Scott	Austin Hospital, Melbourne
Victor Kalff	Alfred Hospital, Melbourne
George Garas	Sir Charles Gairdner Hospital, Perth
Anthony Dowling	St Vincent's Hospital, Melbourne
Darrell Crawford	Princess Alexandra Hospital, Brisbane
John Ring	Flinders Medical Centre, Adelaide
Russell Basser	Royal Melbourne Hospital, Melbourne
Andrew Strickland	Monash Medical Centre, Melbourne
Graeme Macdonald	University of Queensland, Brisbane
Michael Green	Western Hospital, Melbourne
Anna Nowak	NHMRC Clinical Trials Centre, University of Sydney
Blair Dickman	NHMRCClinical Trials Centre, University of Sydney
Haryana Dhillon	NHMRC Clinical Trials Centre, University of Sydney
Val Gebski	NHMRC Clinical Trials Centre, University of Sydney
	
**Principal investigators**	
Paul Thompson	Auckland Hospital, New Zealand
Jonathan Cebon	Austin Hospital, Melbourne, Australia
Michael Boyer	Royal Prince Alfred Hospital, Sydney, Australia
Stuart Roberts	Alfred Hospital, Melbourne, Australia
George Garas	Sir Charles Gairdner Hospital, Perth, Australia
Anthony Dowling	St Vincent's Hospital, Melbourne, Australia
Darrell Crawford	Princess Alexander Hospital, Brisbane, Australia
John Ring	Flinders Medical Centre, Adelaide, Australia
Michael Findlay	University of Auckland, New Zealand
Russell Basser	Royal Melbourne Hospital, Melbourne, Australia
Andrew Strickland	Monash Medical Centre, Melbourne, Australia
Graeme Macdonald	University of Queensland, Brisbane, Australia
Michael Green	Western Hospital, Melbourne, Australia
	
**National Health and Medical Research Council Clinical Trials Centre, University of Sydney**	
Val Gebski and Carol Hargreaves (study statisticians)	
Haryana Dhillon and Blair Dickman (study coordinators)	
Anna Nowak and Martin Stockler (quality of life and clinical epidemiology)	
Burcu Cakir (study manager)	
Rhana Pike (publications editor)	
	
**Trial Steering Committee**	
Jonathan Cebon	Austin Hospital
Michael Findlay	University of Auckland
Haryana Dhillon	NHMRC Clinical Trials Centre
Val Gebski	NHMRC Clinical Trials Centre
Garry Jeffrey	Sir Charles Gairdner Hospital
Graeme Macdonald	University of Queensland
John Ring	Flinders Medical Centre
Steve Riordan	Prince of Wales Hospital
Stuart Roberts	Alfred Hospital
Andrew Scott	Austin Hospital
Martin Stockler	NHMRC Clinical Trials Centre

## Appendix A

Table A1
